# Pathology of fatal *Baylisascaris schroederi* infection in a wild giant panda

**DOI:** 10.1051/parasite/2025026

**Published:** 2025-06-11

**Authors:** Lingling Chang, Danhui Zhang, Yashi Wang, Zun Ren, Yaping Wu, Qiang Zhang, Guanghui Zhao, Guanglin Pan, Xinglong Wang, Xiaomin Zhao, Dewen Tong

**Affiliations:** 1 College of Veterinary Medicine, Northwest A&F University No. 22 Xinong Road Yangling Shaanxi Province 712100 PR China; 2 Qinling Giant Panda Research Center No. 233 Xiguanzheng Street Xi’an City Shaanxi Province 710003 PR China

**Keywords:** Giant panda, *Baylisascaris schroederi*, Parasitic infection, Pathology

## Abstract

*Baylisascaris schroederi* McIntosh, 1939 (Ascarididae), a nematode specific to giant pandas (*Ailuropoda melanoleuca*), is a major health threat, particularly to wild populations. A 20-year-old wild adult female giant panda rescued from a Chinese nature reserve died with a 2-month history of emaciation and weakness. Necropsy was performed. Grossly, the giant panda was very thin with minimal fat stores throughout, and marked serous atrophy of fat around the kidneys. Mesenteric edema was very pronounced in the posterior intestine. The abdominal cavity contained approximately 5 L of orange-yellow, translucent, low-viscosity fluid. There were ca. 1,660 robust ascarids occupying the lumen of the esophagus, stomach, and intestines. Microscopically, the intestine showed moderate necrotizing and eosinophilic enteritis with adult nematodes, consistent with an ascarid. PCR and sequencing confirmed that the ascarid species was *B. schroederi*. This case highlights a fatal *B. schroederi* infection in a wild giant panda, with malnutrition and possible multiple organ failure identified as the primary causes of death.

## Abstract (fr)

*Baylisascaris schroederi* McIntosh, 1939 (Ascarididae), un nématode spécifique des pandas géants (*Ailuropoda melanoleuca*), constitue une menace sanitaire majeure, en particulier pour les populations sauvages. Une femelle panda géant sauvage adulte de 20 ans, sauvée d’une réserve naturelle chinoise, est décédée après deux mois d’émaciation et de faiblesse. Une autopsie a été pratiquée. L’examen macroscopique a révélé que le panda géant était très maigre, avec des réserves adipeuses minimales et une atrophie séreuse marquée de la graisse autour des reins. Un œdème mésentérique était très prononcé dans l’intestin postérieur. La cavité abdominale contenait environ 5 L de liquide jaune orangé, translucide et de faible viscosité. Environ 1 660 ascarides robustes occupaient la lumière de l’œsophage, de l’estomac et des intestins. Au microscope, l’intestin présentait une entérite nécrosante et éosinophile modérée à nématodes adultes, compatible avec un ascaride. La PCR et le séquençage ont confirmé que l’espèce d’ascaride était *B. schroederi*. Ce cas met en évidence une infection mortelle à *B. schroederi* chez un panda géant sauvage, la malnutrition et une possible défaillance multiviscérale ayant été identifiées comme causes principales du décès.

## Introduction

The giant panda (*Ailuropoda melanoleuca*), a symbol of wildlife conservation, is endemic to China. Despite conservation efforts, parasitic diseases remain a significant challenge, particularly for wild populations [9, 13, 16]. Among these, *Baylisascaris schroederi* McIntosh, 1939 is the most prevalent and pathogenic parasite in giant pandas [2, 3, 7, 14]. Infection rates range from 7.1% to 100% in wild and captive individuals [12, 15]. Unlike captive pandas, which benefit from routine deworming and veterinary care, wild pandas are exposed to higher environmental parasite loads and lack access to intervention. Heavy infections with *B. schroederi* can result in malnutrition, systemic complications, and death [6, 7]. This report describes a fatal case of *B. schroederi* infection in a wild giant panda, providing insights into the pathology and implications of severe parasitism.

## Material and methods

### Ethics

The study is a clinical case report, and the animal was submitted for necropsy; hence no ethical committee approval was requested. Informed consent was obtained from the owner of the animal.

### Clinical history

In September 2023, a 20-year-old wild adult female giant panda was discovered in a state of depression in a nature reserve in Shaanxi Province, China. The panda was subsequently transferred to the nearby Giant Panda Research Center for treatment. Clinically, the animal weighed 50 kg (normal weight range: 80 kg–110 kg in healthy wild adult giant panda) with signs of anorexia, emaciation, and weakness. The teeth were badly worn and the upper canine teeth had fallen out.

Abdominal ultrasound revealed significant ascites. The serum chemistry abnormalities primarily showed decreased albumin (20.7 g/L; reference range: 35–53 g/L) and cholinesterase (291 U/L; reference range: 4,000–11,000 U/L), and increased levels of aspartate aminotransferase (AST) (162 U/L; reference range: 0–38 U/L), alanine transaminase (ALT) (74 U/L; reference range: 0–38 U/L), lactic dehydrogenase (LDH) (1,319 U/L; reference: 103–227 U/L), and hydroxybutyrate dehydrogenase (HBDH) (1,131 U/L; reference: 72–182 U/L). A complete blood count was carried out, which revealed no significant abnormalities, with the exception of slightly elevated platelet levels. Fecal flotation revealed ascarid eggs. Molecular testing was negative for canine distemper virus and parvovirus.

The giant panda was given medical treatment (albendazole, dexamethasone, cefradine and antondine) and other supportive care, but her condition continued to deteriorate and she died 2 months after rescue.

### Necropsy and histopathological examination

Necropsy was performed on the day the animal died. The organs with abnormal changes were photographed, and tissue samples were cut and placed in 10% neutral formalin for fixation and paraffin sectioning. The adult ascarids were removed from the intestinal lumen and fixed in 75% alcohol. The fixed tissue samples were embedded in paraffin, sectioned, and stained with hematoxylin-eosin stain, then observed under the microscope and photographed.

### PCR detection

The species of ascarid was determined by PCR, as previously described [14]. DNA was extracted from a single adult nematode body and amplified by PCR using primers targeting the mitochondrial cytochrome oxidase subunit II (COII) gene.

## Results

**Figure 1:**
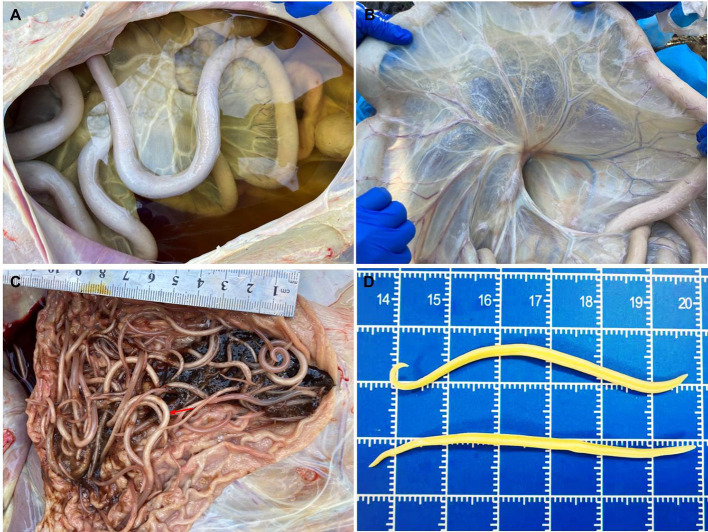
Gross findings in the giant panda. A. Massive orange-yellow transparent fluid in the abdominal cavity. B. Mesentery showing marked edema with gelatinous appearance. C. Ascarids, *Baylisascaris schroederi*, (red arrow) in the lumen of the stomach. D. Adult ascarids, *B. schroederi*, removed from the intestinal lumen, 6–7 cm long and 3 mm wide: the one with the curved tail at the top is the male, and the one with the upright tail at the bottom is the female.

At necropsy, the giant panda was in poor body condition. She was very thin with minimal fat stores throughout, and marked serous atrophy of fat around the kidneys. The abdominal cavity contained approximately 5 L of orange-yellow, translucent, low-viscosity fluid (Fig. 1A). Mesenteric edema was very pronounced in the posterior intestine (Fig. 1B). There were approximately 1,660 robust ascarids occupying the lumen of the esophagus, stomach, and intestines (Figs. 1C–1D).

**Figure 2:**
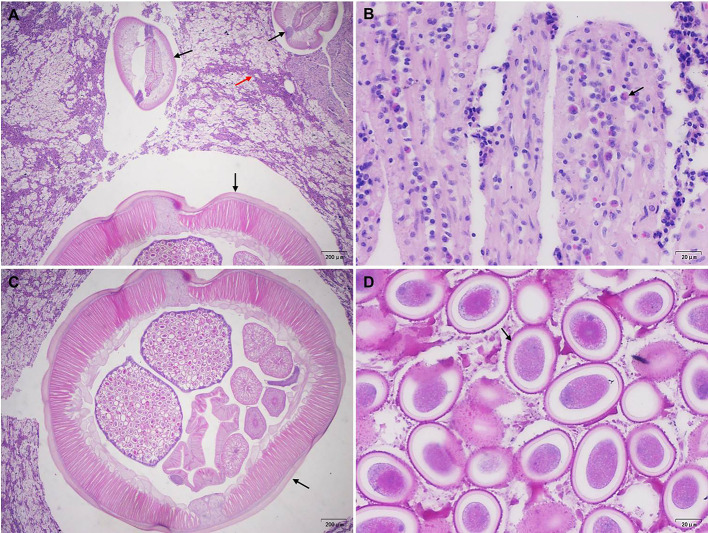
Histopathological changes and nematodes in the giant panda. A. Large amounts of cellular debris and mucus on the surface of the intestinal mucosa (red arrow), mixed with multiple cross-sections of adult nematodes, *Baylisascaris schroederi*, (black arrow) ranging in diameter from 460–500 μm to 2,100–2,300 μm. B. Extensive epithelial necrosis and infiltrates of eosinophils (black arrow) in the lamina propria. C. Cross-section of an adult female nematode (black arrow). D. Oval eggs (black arrow) about 40 μm in diameter filling in the uterus of female nematode.

Microscopically, the intestine showed moderate necrotizing and eosinophilic enteritis with adult nematodes, consistent with ascarids (Figs. 2A–2B). Nematode diameter ranged from 460–500 μm to 2,100–2,300 μm, and the body showed lateral chords and alae, as well as an intestine lined by uninucleate brush-bordered columnar cells (Fig. 2C). Within the female nematode, the uterus was filled with a substantial number of oval eggs measuring approximately 40 μm in diameter (Fig. 2D). No evidence of visceral larval migration was observed on the lung, liver, spleen, or heart sections.

PCR specifically amplified a 270-bp long fragment of the COII gene. Subsequent sequencing (GenBank accession number PV437593) showed 99% identity with GenBank giant panda *Baylisascaris schroederi* isolates.

## Discussion

In this case, *B. schroederi* infection was diagnosed based on clinical examination, pathological changes, and molecular detection.

*Baylisascaris schroederi* is a member of the Ascaridae (Nematoda). In 1939, *B. schroederi* was described in a giant panda brought to the New York Zoo (New York, NY, USA) from China [1]. Besides *B. schroederi*, there are other relatively host-specific *Baylisascaris* species, including *B. procyonis* (raccoons), *B. melis* (badgers), *B. columnaris* (skunks), *B. laevis* (woodchuck), *B. devosi* (fishers and martens), and *B. transfuga* (bears) [6].

The giant panda is the definitive host of *B. schroederi* and sheds infective eggs in feces. The *B. schroederi* eggs can directly infect giant panda without intermediate hosts [1]. One study showed that the prevalence and intensity of *B. schroederi* infection were 52.3% (101/193) and 89 eggs/g of feces, respectively, among the wild giant pandas in Shaanxi Foping Nature Reserve, China [7]. In this case, the wild giant panda was probably exposed to panda feces and ingested infective eggs in its habitat.

Like other *Baylisascaris* species, the life cycle of *B. schroederi* may also undertake liver-lung migration and re-enter the intestinal lumen to develop into mature adults, leading to mechanical and metabolic burden on the host [5, 10, 11, 13]. This case featured an unusually high worm burden (1,660 nematodes), far exceeding the typical range of 1–619 worms reported in previous cases [11]. The possible cause is ingestion of large quantities of eggs from the environment in a short period of time. In addition, deworming is impractical for giant pandas in the wild. High numbers of worms lead to intestinal obstruction and reduced nutrient absorption, malnutrition (serous fat atrophy), and possible multiple organ failure, as seen in this case.

Previous reports have documented that eosinophilic and granulomatous inflammation are typical features of parasite infection. Eosinophilic infiltrates were seen in the intestinal lamina propria in this case. However, no inflammatory lesions were found in extra-intestinal tissues. Potentially, all *Baylisascaris* spp. can cause similar visceral larval migration lesions commonly found in the lungs and liver [2–4]. One report showed a rare case in which *B. schroederi* reached the pancreas and led to fatally acute pancreatitis in a giant panda [8]. However, no histological evidence of visceral larval migration was observed in this case.

## Conclusion

This report highlights the profound pathological impact of *B. schroederi* in wild giant pandas. The high parasite load described here underscores the challenges of controlling parasitic diseases in wild populations. Improved monitoring, habitat management, and targeted intervention strategies are essential to mitigate the impact of parasitic diseases on giant panda conservation.
